# Exploring New Links Among Keratoconus, Hormonal Factors, and Medications: Insights From a Case–Control Study Utilizing the All of Us Database

**DOI:** 10.1167/tvst.13.11.18

**Published:** 2024-11-18

**Authors:** Carol Beatty, Amy Estes, Hongyan Xu, Yutao Liu

**Affiliations:** 1 Medical College of Georgia, Augusta University, Augusta, GA, USA; 2 Department of Ophthalmology, James and Jean Culver Vision Discovery Institute, Augusta University, Augusta, GA, USA; 3 Department of Biostatistics, Data Science and Epidemiology, Augusta University, Augusta, GA, USA; 4 Department of Cellular Biology and Anatomy, Center for Biotechnology and Genomic Medicine, James and Jean Culver Vision Discovery Institute, Augusta University, Augusta, GA, USA

**Keywords:** keratoconus, hormone, estrogen, vitamin C, tetracycline

## Abstract

**Purpose:**

We aimed to identify clinical factors associated with keratoconus (KC) risk in the All of Us database.

**Methods:**

This retrospective matched case–control study utilized patient data from the All of Us Research Program. All patients with a KC diagnosis (*n* = 572) were enrolled in the study and matched with three controls (*n* = 1716) based on age ± 1 year, race, ethnicity, and sex. The patients’ medical histories, including diabetes, sleep apnea, obesity, smoking, ocular surface disease (encompassing dry eye, eczema, and allergic or atopic conjunctivitis), allergic rhinitis, pregnancy, estrogen-containing medications, tetracyclines, and vitamin C supplementation, were collected using electronic health records. Multivariable odds ratios (ORs) between KC and health history were calculated using the R programming language.

**Results:**

The study included 2288 participants with an average age of 58.7 years. All included variables showed a significant positive correlation with KC except smoking history, which showed a negative correlation. The most significant correlations were ocular surface disease (OR = 6.04) and obesity (OR = 1.82). Significant positive associations were also identified for tetracyclines and estrogen-containing medications with KC. Smoking was negatively correlated.

**Conclusions:**

In addition to previously known risk factors, patients with a history of increased estrogen exposure and tetracycline usage were more likely to have a KC diagnosis whereas those with a smoking history were less likely.

**Translational Relevance:**

Understanding the risk factors for KC, including estrogen exposure and tetracycline medications, enhances our ability to identify at-risk patients and implement earlier screening, diagnosis, and interventions.

## Introduction

Keratoconus (KC) is a bilateral and asymmetric corneal ectasia affecting >300,000 people in the United States.^1^^–^^4^ This progressive condition is characterized by irregular thinning and steepening of the cornea, leading to a conical shape.^5^ These changes to the cornea result in irregular astigmatism and diminished visual acuity,^6^ both of which negatively impact patients’ quality of life.^7^ KC is most commonly diagnosed during the second or third decade of life, thus imposing both financial and social burdens at a relatively early age.^8^^,^^9^

Due to the progressive nature of the disease, early diagnosis and treatment can improve visual outcomes.^10^ KC is a multifactorial disease influenced by both environmental and genetic factors.^11^ The disease is highly associated with eye rubbing, and many conditions are correlated with eye rubbing such as dry eye, allergy, and conjunctivitis.^12^^–^^15^ Eye rubbing has been hypothesized to be causative, as rubbing one's eye can mechanically thin out the cornea.^16^ Through this study, we aimed to widen our understanding of the known risk factors for KC, including eye rubbing, atopy, allergy, and eczema.^8^^,^^17^^–^^19^

Identification of known risk factors for KC might increase clinical suspicion and result in earlier evaluation of the condition.^20^^–^^22^ In this work, we sought to identify new risk factors that might assist in early diagnosis or detection of KC progression. Correlations between KC and pregnancy have been hypothesized but are lacking sufficient evidence to this point.^18^^,^^23^^,^^24^ In addition to investigating this hypothesized association between KC and pregnancy in a larger dataset than previously reported, we expanded our analysis to evaluate a potential link between the ocular condition and women's hormones. Vitamin C is an essential cofactor of enzymes involved in collagen synthesis and crosslinking, having been widely used to stimulate corneal stromal fibroblast cells to secrete extracellular matrix in in vitro cell culture experiments.^25^^,^^26^ These same enzymes are thought to play a role in corneal remodeling and define the initiation and progression of KC.^27^ Notably, levels of vitamin C were found to be elevated in KC corneas^28^ and increased following corneal cross-linking.^29^ Vitamin C deficiency has also been hypothesized to correlate with KC.^30^ Considering the mixed body of literature surrounding this topic, we investigated whether a relationship between vitamin C supplementation and the prevalence of KC existed in our study population. Although KC is a non-inflammatory disease, more evidence has indicated the potential involvement of inflammatory factors in KC pathogenesis.^31^^,^^32^ Antibiotics, such as tetracyclines, are often prescribed during inflammatory ocular events such as ocular rosacea or sterile corneal ulcerations.^33^^–^^35^ We were interested in exploring whether tetracyclines, some of the most commonly used ocular antibiotics, are associated with KC risk.^33^

The study was conducted utilizing the All of Us research database. The All of Us Research Program is a National Institute of Health–funded data initiative to collect health data on more than 1 million Americans. The database collects patient-level data on a diverse population from across the United States.^36^ The platform allows us to analyze a large, diverse population to understand better the relationships between a relatively rare ocular disease and common risk factors.

## Methods

This was a retrospective case–control study utilizing the data available in the All of Us database. The database includes patients 18 years and older recruited within the period of May 2018 to January 2022. In the All of Us platform, volunteers may complete health-related surveys and consent to sharing their electronic health records (EHRs). The health data of each participant were transferred to the database; they were then deidentified and made accessible to registered users for research purposes.^37^ Data used in this study are available to registered researchers of the All of Us Researcher Workbench. For information about access, please visit https://www.researchallofus.org/.

This study selected two cohorts of adult patients with associated EHRs using the All of Us data analysis workbench: KC patients and matched non-KC patients. The KC patients were identified by the Systemized Nomenclature of Medicine, Clinical Terms (SNOMED-CT) term “keratoconus,” based on the International Classification of Diseases (ICD) codes for KC. The KC cohort was built to include all patients with at least one KC diagnosis in their EHRs (*n* = 572). Age, ethnicity, and sex data were extracted from the survey data available in the database. Participants in the KC cohort were matched with three controls based on age ± 1 year, ethnicity, and sex to create the matched non-KC cohort (*n* = 1716).

ICD codes were used to identify covariates, including a history of diabetes, sleep apnea, obesity, dry eye, eczema, allergic rhinitis, allergic or atopic conjunctivitis, tetracycline medication, vitamin C supplementation, pregnancy, and estrogen-containing medication. History of smoking was identified using a combination of survey data and ICD codes. Participants who reported smoking more than 100 cigarettes in their lifetime, smoking for more than 5 years, or smoking a cigarette in the last month were defined as smokers by the survey results. Due to significant overlap in the pathophysiology and previously reported strong evidence for dry eye, allergic or atopic conjunctivitis, and eczema, all participants with these risk factors were grouped into one category referred to as “ocular surface disease.”

Advanced statistical techniques were employed within the All of Us researcher workbench to analyze the collected data. Data analysis was conducted on the Jupyter Notebook web-based platform using the R programming language (R Foundation for Statistical Computing, Vienna, Austria). Tests were performed using the “oddsratio()” function from the “epitools” package and “glm()” function from the “stats” package. Univariable odds ratios (ORs) were calculated comparing KC and each collected element of the participants’ health histories (ocular surface disease, diabetes, sleep apnea, obesity, smoking, allergic rhinitis, pregnancy, estrogen-containing medications, tetracyclines, and vitamin C supplementation). A logistic regression was performed using all variables to show significant correlations to KC (ocular surface disease, diabetes, sleep apnea, obesity, smoking, allergic rhinitis, pregnancy, estrogen-containing medications, tetracyclines, and vitamin C supplementation) as covariates to calculate multivariable ORs. Also, 95% confidence intervals (CIs) and *P* values were additionally calculated. All analyses exploring the history of pregnancy and estrogen-containing medication included only female KC participants (*n* = 316) and their matched controls (*n* = 948).

## Results

The analyzed cohorts included 572 patients with KC and 1716 patients without KC. The mean ages of participants were 58.7 ± 15.6 years for KC cases and 58.7 ± 15.6 years for controls. In both the KC and control cohorts, 55% of participants were female and 25% were Hispanic. In the KC cohort, 48% of participants were European American, 23% were Black or African American. In the control cohort, 51% were European American and 18% were Black or African American (Table 1).

**Table 1.** Demographics of the Study Populations

	KC (*n* = 572)	Non-KC (*n* = 1716)	*P*
Age, mean ± SD	58.7 ± 15.6	58.7 ± 15.6	1.0
Sex, *n* (%)			1.0
Female	316 (55)	948 (55)	
Male	246 (43)	738 (43)	
Other	10 (2)	30 (2)	
Ethnicity, *n* (%)			1.0
Not Hispanic or Latino	405 (71)	1215 (71)	
Hispanic or Latino	144 (25)	432 (25)	
Unknown	23 (4)	69 (4)	
Race, *n* (%)			>0.99
White	272 (48)	877 (51)	
Black or African American	129 (23)	315 (18)	
Asian	8 (1)	42 (2)	
Middle Eastern or North African	6 (1)	7 (0)	
Native Hawaiian or Other Pacific Islander	1 (0)	3 (0)	
More than one population	8 (1)	27 (2)	
Unknown	148 (26)	445 (26)	

The strongest correlation to KC in the multivariable analysis was ocular surface disease (57.87%; OR = 6.04; 95% CI, 4.77–7.65). Additional variables showing a statistically significant correlation to participants with a diagnosis of KC in comparison to matched controls when accounting for all other tested variables were obesity (44.76%; OR = 1.82; 95% CI, 1.40–2.37), history of estrogen-containing medication (44.30%; OR = 1.77; 95% CI, 1.25–2.51), and tetracycline usage (30.59%; OR = 1.36; 95% CI, 1.03–1.79). In contrast, participants with KC were statistically significantly less likely to have a history of smoking (31.82%; OR = 0.64; 95% CI, 0.51–0.81) in the multivariable analysis. All other variables tested showed a positive correlation with KC but were not statistically significant: sleep apnea (32.52%; OR = 1.34; 95% CI, 1.00–1.80); type II diabetes (29.37%; OR = 1.33; 95% CI, 0.99–1.77); vitamin C supplementation (19.75%; OR = 1.31; 95% CI, 0.95–1.80); pregnancy (23.73%; OR = 1.30; 95% CI, 0.87–1.94); allergic rhinitis (33.57%; OR = 1.22; 95% CI, 0.93–1.61); and type I diabetes (4.72%; OR = 1.00; 95% CI, 0.52–1.95) (Table 2, Fig.).

Results from the univariable analysis are included in Table A1 which is available in Appendix A. Additionally, results of the univariable (Table B1) and multivariable (Table B2) analyses without grouping dry eye, eczema, and allergic or atopic conjunctivitis are included in Appendix B.

**Table 2.** Multivariable Analysis of Clinical Factors Associated With KC

Variable	Multivariable OR	95% CI	*P*
Ocular surface disease^a^	6.04	4.77–7.65	<0.0001
Obesity	1.82	1.40–2.37	<0.0001
Estrogen-containing medication	1.77	1.25–2.51	0.00139
Tetracycline	1.36	1.03–1.79	0.02945
Sleep apnea	1.34	1.00–1.80	0.05247
Type II diabetes	1.33	0.99–1.77	0.05884
Vitamin C supplementation	1.31	0.95–1.80	0.09878
Pregnancy	1.30	0.87–1.94	0.2025
Allergic rhinitis	1.22	0.93–1.61	0.1465
Type I diabetes	1.00	0.52–1.95	0.9923
Smoking	0.64	0.51–0.81	0.00163

a Ocular surface disease includes participants with dry eye, allergic or atopic conjunctivitis, or eczema.

**Figure.**
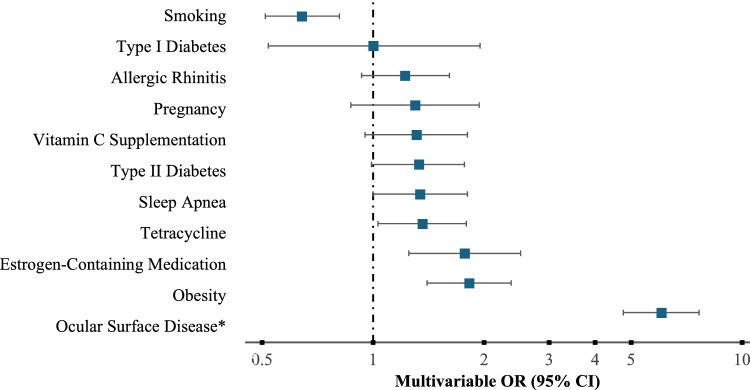
Multivariable odds ratios of the association between keratoconus and health history. *Ocular surface disease includes participants with dry eye, allergic or atopic conjunctivitis, or eczema.

## Discussion

Our study identified novel risk factors associated with KC (estrogen-containing medications and tetracycline usage), confirmed known risk factors (ocular surface disease and obesity), and provided evidence of possible protective effects of smoking against KC. This analysis revealed a correlation between estrogen and KC. A history of estrogen-containing medications was significantly associated with KC even when accounting for covariates, and previous pregnancy showed a positive correlation although it was not significant. Several case studies have detailed instances of progression and worsening of KC following pregnancy^23^^,^^24^^,^^38^^,^^39^; however, our KC cohort included 316 female participants, which allowed for wide-scale correlation analysis. The most common estrogen-containing medication reported in our cohorts was oral contraceptive pills. Several studies have suggested a relationship between estrogen and the ocular disease, finding increased estrogen receptors in KC corneas.^40^^,^^41^ This mechanism might offer a basic explanation for the 1.30× odds that KC patients have a history of pregnancy and 1.77× odds of previous estrogen-containing medication use.

The history of tetracycline usage was interestingly found to correlate positively with KC in both the univariable and multivariable analyses. Tetracyclines are most commonly used as a broad-spectrum antibiotic, but the drug class has shown non-antimicrobial properties. Tetracyclines have known anti-inflammatory effects, inhibiting matrix metalloproteinase (MMP) and interleukin-1 (IL-1) production.^42^ The reported correlation to KC could relate to the usage of tetracycline as an ocular anti-inflammatory, prescribed in instances of corneal ulceration, ocular rosacea, keratoconjunctivitis sicca, other dermatological conditions, or even dry eye.^33^^,^^35^^,^^42^^,^^43^ Tetracyclines are thought to slow pathological soft-tissue breakdown through anti-collagenase activity, leading to its use in sterile corneal ulcers.^33^^,^^44^ Alternatively, the correlation could possibly be attributed to antibiotic management of KC treatments and sequelae. Tetracyclines and other ocular antibiotics are rarely prescribed in the event of acute corneal hydrops, a rare complication of KC, or following corneal collagen cross-linking, a procedure shown to slow KC progression.^45^^–^^47^ Future work is needed to better understand if the association between KC and tetracyclines is a direct risk factor altering corneal tissue, secondary to underlying inflammatory diseases, or is due to an alternative explanation.

History of smoking was the only covariate with a negative correlation with KC. Current literature is split on smoking, with some studies finding a positive association, some a negative association, and some no association at all.^48^^–^^52^ Our study, with a population of 572 KC patients, analyzed a larger KC population than previous studies exploring the relationship between smoking and KC.^48^^–^^52^ A proposed hypothesis relating smoking to KC is its impact on the meibomian glands, resulting in a dry eye and increasing friction across the cornea.^48^ However, an alternative hypothesis could explain the mechanism by which smoking showed a negative correlation and possible protective factor against KC in our study. Smoking results in reactive glycation products, nitrogen oxide, and nitrites, which cross-link and modify collagen proteins. These modifications could contributed to corneal stabilization, slowing the pathogenesis of KC.^51^

Our work additionally confirmed previous studies, which have established known risk factors associated with KC including dry eye,^14^^,^^15^ allergy,^8^^,^^12^^,^^13^^,^^17^ eye rubbing,^8^^,^^12^^,^^13^^,^^16^^,^^17^^,^^19^ eczema,^8^^,^^19^ and obesity.^53^^,^^54^ Ocular surface disease, encompassing dry eye, allergic or atopic conjunctivitis, and eczema, was most strongly associated with KC. All three conditions are strongly associated with eye rubbing and proposed to contribute to the development or progression of KC through direct mechanical injury to the cornea.^8^^,^^12^^,^^13^^,^^55^ Additional evidence suggests inflammation might also be contributing to this strong correlation.^15^ Pro-inflammatory cytokines, including IL-1, IL-6, interferon‐gamma (IFN-γ), and tumor necrosis factor alpha (TNF-α), are known to be elevated in KC tears, indicating an inflammatory microenvironment on the surface of KC corneas.^56^^,^^57^ Eye rubbing and allergic eye disease have similarly been shown to increase ocular inflammatory markers, including IL-1, IL-6, MMP, and TNF-α.^58^^,^^59^ The pathophysiology explaining the correlation of KC with obesity is not entirely understood, but it is hypothesized that obesity leads to weaker eyelid skin due to decreased elastin in the tarsal plate. This subsequently reduces the ability of the eyelid to protect the cornea and increases mechanical injury to the cornea.^53^ Another hypothesis suggests that the relationship of obesity to KC is due to the systemic effects characteristic of chronic metabolic inflammation.^54^ Our confirmation of previous findings not only adds to the body of evidence but also helps to validate our population, as it broadly aligns with current literature.

Our population was accessed through the All of Us database. This allowed for the analysis of a relatively large dataset of patients with and without KC who were representative of patients in the United States.^36^ Considering the rarity of KC, the sample size and diversity were significant strengths of the study, a major difference that contributed to the unique findings of our study compared to those of the published literature. Previously reported risk factors were analyzed in a large, diverse database, and evidence of novel risk factors (i.e., estrogen-containing medication) was identified in a large, clinical case–control study (*n* = 2288) rather than in a case series or in studies using animal models. Our results are limited by several factors, however. This work was a retrospective cross-sectional study. The analysis offers strong evidence of the correlation between KC and its risk factors but does not examine causality. The database does not allow for determination of the stage or severity of KC. Controls were determined by a lack of KC diagnosis, so it is possible that individuals with undiagnosed KC were included in the cohort. Alternatively, a coding error in the health record could allow patients without diagnosis to be included in our KC cohort. Additionally, the average age of participants in the KC cohort was 58.7 years. Because onset of the disease typically occurs in the second or third decade of life, it is likely that these patients have established, non-progressive KC.^8^^,^^9^ Some interventions, such as medication or supplementation, might be less relevant due to the static nature of KC in the studied population. Our analysis did not account for any temporal relationship between risk factors and KC diagnosis.

In conclusion, we determined that a history of estrogen-containing medication and tetracycline usage increases the odds of KC but a history of smoking decreases the odds, even when accounting for ocular surface diseases and obesity as covariates. Because a diagnosis of KC is most common early in life, carries a significant burden on quality of life, and has effective treatment options, providers should be aware of all disease risk factors to aid in early diagnosis and intervention.

## Appendix A. Appendix A

**Table A1.** Univariable Analysis of Clinical Factors Associated With KC

Variable	Univariate OR	95% CI	*P*-Value
Pregnancy	2.32	1.67–3.21	<0.0001
Estrogen-Containing Medication	3.64	2.76–4.80	<0.0001
Type I Diabetes	3.35	1.92–5.86	<0.0001
Type II Diabetes	2.67	2.13–3.35	<0.0001
Sleep Apnea	3.53	2.81–4.43	<0.0001
Obesity	3.5	2.86–4.30	<0.0001
Tetracycline Usage	2.98	2.37–3.74	<0.0001
Vitamin C Supplementation	2.77	2.11–3.62	<0.0001
Smoking	0.713	0.582–0.870	0.0009
Allergic Rhinitis	3.28	2.62–4.10	<0.0001
Ocular Surface Atopy	8.39	6.78–10.42	<0.0001

CI, confidence interval; OR, odds ratio; KC, keratoconus.

* Ocular Surface Disease includes participants with Dry Eye, Allergic or Atopic Conjunctivitis, or Eczema.

## Appendix B. Appendix B

**Table B1.** Univariable Analysis of Clinical Factors Associated With KC

Variable	Univariable OR	95% CI	*P*-Value
Pregnancy	2.32	1.67–3.21	<0.0001
Estrogen-Containing Medication	3.64	2.76–4.80	<0.0001
Type I Diabetes	3.35	1.92–5.86	<0.0001
Type II Diabetes	2.67	2.13–3.35	<0.0001
Sleep Apnea	3.53	2.81–4.43	<0.0001
Obesity	3.5	2.86–4.30	<0.0001
Tetracycline Usage	2.98	2.37–3.74	<0.0001
Vitamin C Supplementation	2.77	2.11–3.62	<0.0001
Smoking	0.713	0.582 – 0.870	0.0009
Dry Eye	10.43	7.95–13.80	<0.0001
Eczema	4.01	3.14–5.11	<0.0001
Allergic Rhinitis	3.28	2.62–4.10	<0.0001
Allergic or Atopic Conjunctivitis	6.69	4.54–10.02	<0.0001

CI, confidence interval; OR, odds ratio.

**Table B2.** Multivariable Analysis of Clinical Factors Associated With KC

Variable	Multivariable OR	95% CI	*P*-Value
Pregnancy	1.37	0.905–2.07	0.133
Estrogen-Containing Medication	1.88	1.30–2.70	0.000678
Type I Diabetes	1.13	0.567–2.23	0.734
Type II Diabetes	1.28	0.950–1.72	0.101
Sleep Apnea	1.18	0.868–1.60	0.287
Obesity	1.98	1.51–2.59	<0.0001
Tetracycline Usage	1.6	1.20–2.12	0.00108
Vitamin C Supplementation	1.37	0.983–1.89	0.06066
Smoking	0.614	0.484–0.777	<0.0001
Dry Eye	7.19	5.35–9.72	<0.0001
Eczema	1.92	1.43–2.58	<0.0001
Allergic Rhinitis	1.29	0.970–1.70	0.0777
Allergic or Atopic Conjunctivitis	3.21	2.05–5.07	<0.0001

CI, confidence interval; OR, odds ratio; KC, keratoconus.
